# Plant peroxisome proteostasis—establishing, renovating, and dismantling the peroxisomal proteome

**DOI:** 10.1042/EBC20210059

**Published:** 2022-08-05

**Authors:** DurreShahwar Muhammad, Kathryn A. Smith, Bonnie Bartel

**Affiliations:** Department of BioSciences, Rice University, Houston, TX 77005, U.S.A.

## Abstract

Plant peroxisomes host critical metabolic reactions and insulate the rest of the cell from reactive byproducts. The specialization of peroxisomal reactions is rooted in how the organelle modulates its proteome to be suitable for the tissue, environment, and developmental stage of the organism. The story of plant peroxisomal proteostasis begins with transcriptional regulation of peroxisomal protein genes and the synthesis, trafficking, import, and folding of peroxisomal proteins. The saga continues with assembly and disaggregation by chaperones and degradation via proteases or the proteasome. The story concludes with organelle recycling via autophagy. Some of these processes as well as the proteins that facilitate them are peroxisome-specific, while others are shared among organelles. Our understanding of translational regulation of plant peroxisomal protein transcripts and proteins necessary for pexophagy remain based in findings from other models. Recent strides to elucidate transcriptional control, membrane dynamics, protein trafficking, and conditions that induce peroxisome turnover have expanded our knowledge of plant peroxisomal proteostasis. Here we review our current understanding of the processes and proteins necessary for plant peroxisome proteostasis–the emergence, maintenance, and clearance of the peroxisomal proteome.

## Introduction

Harvesting energy, orchestrating development, and adapting to environmental changes are critical to organismal success—peroxisomes contribute to all three processes. Peroxisomes isolate β-oxidation and other oxidative reactions that produce toxic byproducts, including reactive oxygen and nitrogen species (ROS/RNS), and detoxifying these compounds is a conserved peroxisomal function. Peroxisomes are found in most eukaryotes and encompass a unique combination of features found in other organelles. Like several organelles defined by delimiting membranes and lacking DNA, peroxisomes can originate from the endoplasmic reticulum (ER) [1]. Like the nucleus, peroxisomes can import proteins without unfolding [2,3]. Like mitochondria and chloroplasts, peroxisomes can divide by fission [4]. Like multivesicular endosomes, peroxisomes can contain intralumenal vesicles derived from their outer membrane [5]. These sub-peroxisomal structures may operate in fatty acid metabolism [5], a fundamental peroxisomal function. Plants offer distinctive insights into peroxisomal fat processing, as fatty acid β-oxidation is exclusively peroxisomal in plants [6]. In contrast, metazoans split this task between peroxisomes and mitochondria.

Specific peroxisomal reactions vary based on organism, tissue, and developmental stage, which necessitates modification of the peroxisomal proteome to prioritize specialized reactions. Several plant hormones, including jasmonate and auxin, are partially synthesized in peroxisomes. Jasmonates are needed for pathogen responses and fertility (reviewed in [7,8]), and the auxin output from peroxisomes contributes to cotyledon expansion [9], root hair elongation [9], and lateral root development [10]. Peroxisomal catabolism of stored lipids fuels early seedling growth (reviewed in [11]). After seedlings are established, leaf peroxisomes house key steps in photorespiration, which improves photosynthetic efficiency by recycling glycolate to glycerate, allowing fixed carbon to reenter the Calvin cycle (reviewed in [12]). Peroxisome functions return to lipid catabolism during senescence, another transitional stage characterized by coordinated shifts in protein levels [13]. During senescence, peroxisomes also serve as signaling hubs of reactive species that coordinate with other metabolic organelles (reviewed in [14]).

Because plant peroxisomes carry out critical metabolism, peroxisome proteostasis is vital. Peroxisomal biogenesis and maintenance is facilitated by proteins termed peroxins (PEX proteins), which orchestrate biogenesis, division, and protein delivery to the lumen and membranes of the organelle. Peroxisomes are essential in plants; nearly all viable *pex* mutants are partial loss-of-function alleles, as complete loss of most Arabidopsis PEX proteins results in embryonic or gametophytic lethality [15–22]. Even partial peroxin dysfunction can confer serious plant growth defects [23–28].

Peroxisomal proteostasis contributes to peroxisomal specialization in response to developmental and environmental cues and counters the damaging effects of the byproducts of oxidative peroxisomal reactions. Here, we summarize our current understanding of peroxisomal proteostasis in plants and supplement these findings with insights from other models to bridge the gaps in plant studies.

## Emergence of the peroxisomal proteome–transcription, translation, and trafficking

**Figure 1.**
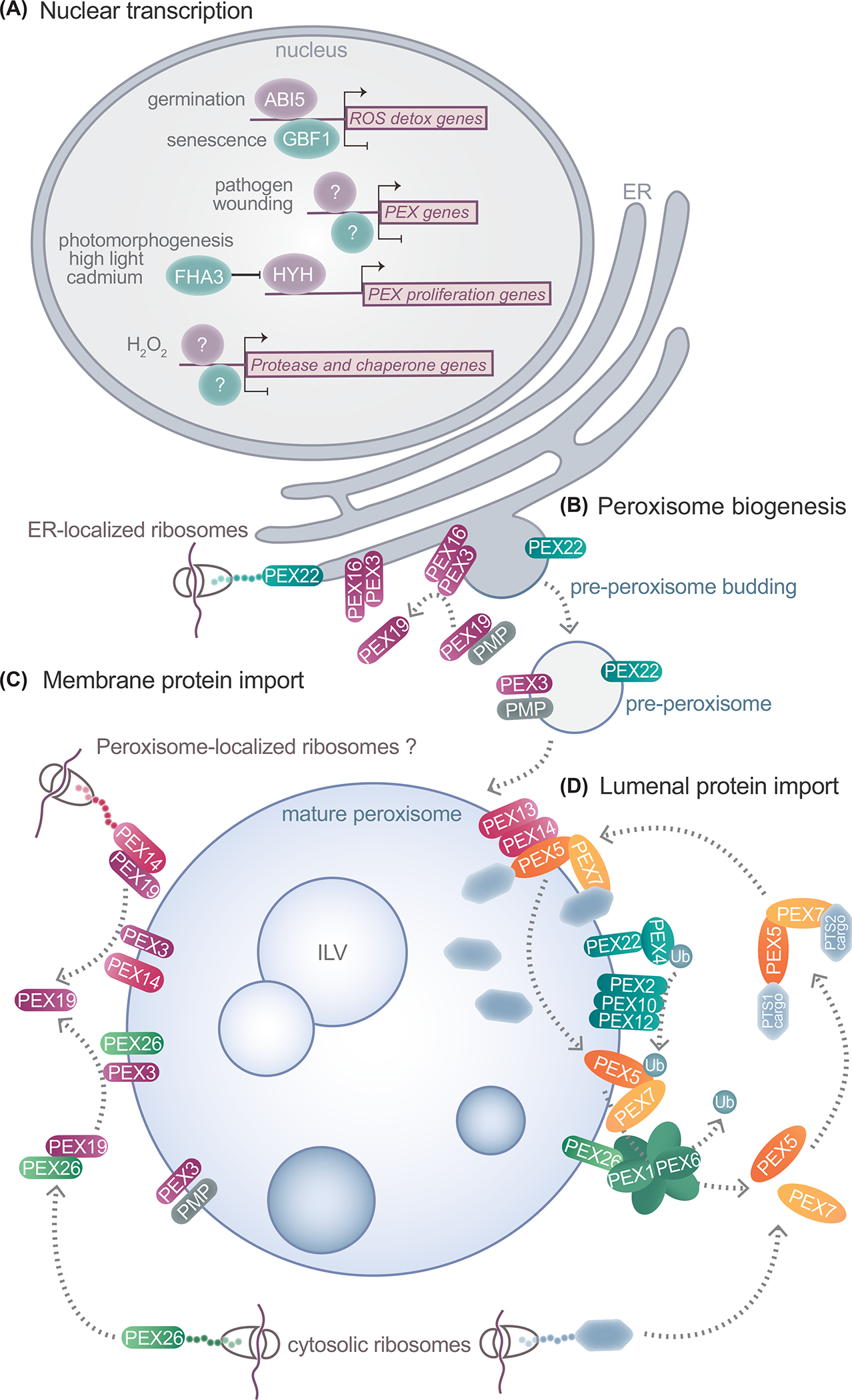
Establishing the peroxisomal proteome (**A**) Nuclear encoded peroxisomal protein genes are governed by various transcriptional activators (mauve) and repressors (teal) and are modulated during development and by abiotic and biotic challenges that elicit individual-protein and whole-organelle changes. (**B**) As part of the endomembrane system, peroxisomes arise from the ER as pre-peroxisomes lacking lumenal proteins. (**C**) mRNA of some peroxisomal membrane proteins (PMPs) are likely translated by ER-localized ribosomes and the protein is trafficked through the ER to bud with pre-peroxisomes. Other PMPs are translated by cytosolic and potentially peroxisome-localized ribosomes. PEX19 acts as a chaperone, binding to the mPTS (membrane peroxisome targeting signal) of PMPs and transferring mPTS-cargo to PEX3 for membrane insertion. (**D**) Mature peroxisomes contain PMPs, intralumenal vesicles (ILVs) derived from the outer membrane, and lumenal proteins. PTS1 and PTS2 (peroxisome targeting signal 1 and 2) cargo (enzymes, chaperones, proteases) are imported into the lumen via receptors (PEX5 and PEX7) and the membrane docking complex (PEX13 and PEX14). After delivery of lumenal cargo, PEX5 is monoubiquitinated by peroxisome-associated ubiquitination machinery (the PEX4-PEX22 ubiquitin conjugating enzyme complex and the PEX2-PEX10-PEX12 ubiquitin ligase enzyme complex) and retrotranslocated for reuse by an ATPase complex (PEX1, PEX6, and PEX26).

Like many stories in biology, the narrative of peroxisomal proteins begins in the nucleus. Peroxisomal proteins are encoded by nuclear DNA; thus, initial regulation takes place outside of the organelle. Transcripts encoding proteins that decompose peroxisomal ROS (e.g., catalase (CAT), glutathione S-transferase, superoxide dismutase, and ascorbate peroxidase) are dynamically expressed in conditions that stimulate ROS production and/or promote peroxisome turnover, including pathogen and wound response, high light stresses, nutrient limitations, metal imbalances, drought stress, and senescence [29–43]. For example, the Arabidopsis ABI5 and GBF1 transcription factors activate *CAT1* during seed germination and inhibit *CAT2* during senescence and pathogen infection, respectively (Figure 1A) [29,44]. Moreover, transcriptional profiling of autophagy mutants and senescing Arabidopsis and maize leaves reveals enrichment in peroxisome-associated gene ontology terms [30,33,36], suggesting that peroxisomal protein levels and reactions are modulated during cellular remodeling.

In addition to transcripts encoding peroxisomal enzymes, transcripts encoding peroxins, which build peroxisomes, are also regulated. Wounding and *Pseudomonas syringae* infection induce Arabidopsis *PEX1*, *PEX5*, *PEX10*, and *PEX14* transcripts (Figure 1A) [35], but the transcriptional regulators of these genes are not identified. PEX11 is implicated in peroxisome proliferation, and Arabidopsis *PEX11* mRNA levels are dynamic. For example, *PEX11A* and *PEX11B* are induced by cadmium and high light, respectively (Figure 1A) [31,39,40]. Phytochrome A (PHYA) and the HYH transcription factor up-regulate *PEX11B* transcription, and *phya* and *hyh* mutants have fewer peroxisomes [31]. HYH directly binds the *PEX11B* promoter to promote peroxisome proliferation during seedling photomorphogenesis [31], and FHA3 negatively regulates *PEX11B* via HYH binding (Figure 1A) [45]. Further elucidation of transcriptional regulators specific to peroxisomal protein genes is needed to fully understand plant peroxisome proteostasis.

In addition to assessing transcriptional regulation following environmental challenges, analysis of transcript changes in mutants can elucidate the importance of specific proteins in peroxisomal processes. Dysfunctional peroxisomes can trigger retrograde signaling from the peroxisome to the nucleus. For example, catalase disruption in Arabidopsis alters expression of genes involved in abiotic and biotic stress response, plant growth regulation, and MAPK cascades [42], implicating ROS regulation in these transcriptional responses. Moreover, impaired peroxisomal protein import in a *Caenorhabditis elegans pex* mutant triggers induction of peroxisomal catalase and Lon protease genes by a ligand-activated transcription factor and a mediator of RNA polymerase II transcription [46]. Additional exploration of transcriptional changes in peroxisome-specific mutants is needed to fully understand this signaling in plants.

Protein delivery to the peroxisome is accomplished by PEX proteins that recognize Peroxisome Targeting Signals (PTSs) on peroxisomal cargo. Peroxisomal membrane proteins (PMPs), including most peroxins, are targeted through an mPTS (membrane PTS) and can be directly inserted into the peroxisome membrane or inserted into the ER before moving to nascent peroxisomes via ER budding. For example, a fluorescent protein tagged with the PEX26 mPTS localizes to peroxisome membranes in Arabidopsis seedlings, whereas the PEX22 mPTS localizes a reporter to both ER and peroxisome membranes [5]. Cytosolic PEX19 binds the mPTS and acts as a chaperone that accompanies the PMP cargo to PEX3, a PMP that assists with membrane insertion [47,48] (Figure 1C).

In addition to PEX3 and PEX19, Arabidopsis PEX16 assists in targeting PMPs that first insert into the ER [49]. Viable *pex16* alleles show enlarged peroxisomes [50], and peroxisomes are enlarged or misshapen when PEX3, PEX16, or PEX19 levels are reduced via RNAi [51]. Exploration of PEX3 and PEX19 functions is complicated by gene duplications resulting in two isoforms of both peroxins in Arabidopsis [20,52]. Whether Arabidopsis *pex3*, *pex16*, or *pex19* mutants display PMP sorting defects has not been examined.

mRNAs from nuclear genes that encode proteins destined for the ER, chloroplasts, and mitochondria can be translated by organelle-associated ribosomes— streamlining protein synthesis and organellar targeting (reviewed in [53]). For example, yeast *PEX3* transcripts are found in ER-associated ribosomes [54]. Although proteins inserted directly into peroxisomes are typically thought to originate from cytosolic ribosomes, peroxisome-specific ribosomal profiling and single-molecule RNA fluorescence *in situ* hybridization in yeast reveals 11 peroxisomal protein transcripts, mostly PMPs, translated by peroxisome-proximal ribosomes [55]. Similarly, transcripts of several yeast peroxisome lumenal proteins are peroxisome-associated [54]. It will be interesting to learn whether localized translation is also associated with plant peroxisomes.

Peroxisomes are unusual among organelles in that they can import fully folded proteins [2,3]. Lumenal protein import relies on one of two peroxisome targeting signals that are recognized by cytosolic receptors (reviewed in [12]). PEX5 binds PTS1 cargo and PEX7 binds PTS2 cargo (Figure 1D). Like sequences targeting proteins to the ER, mitochondria, and chloroplasts, the nonapeptide PTS2 is near the N-terminus and is usually removed after import [56]. In contrast, the C-terminal PTS1 tripeptide [57] is not removed. Cargo-bound PEX5 and PEX7 complex with docking machinery, PEX13 and PEX14, at the peroxisomal membrane, resulting in lumenal delivery (Figure 1D). After cargo delivery, PEX5 is mono-ubiquitinated by dedicated ubiquitination machinery in the peroxisomal membrane to allow retrotranslocation from the membrane by peroxisome-anchored ATPases for further import rounds (Figure 1D).

The presence of PTS1 and PTS2 signals has enabled robust bioinformatic prediction of the peroxisomal proteome (reviewed in [58]), which is supported by proteomic analysis of purified peroxisomes from a variety of tissues (reviewed in [59]). In addition, some peroxisomal proteins are targeted to multiple locations (reviewed in [60]). For example, catalase, although predominantly peroxisomal, may also localize in the nucleus under certain conditions in Arabidopsis [61].

## Maintenance of the peroxisomal proteome–the 26S ubiquitin-proteasome system, chaperones, and proteases

**Figure 2.**
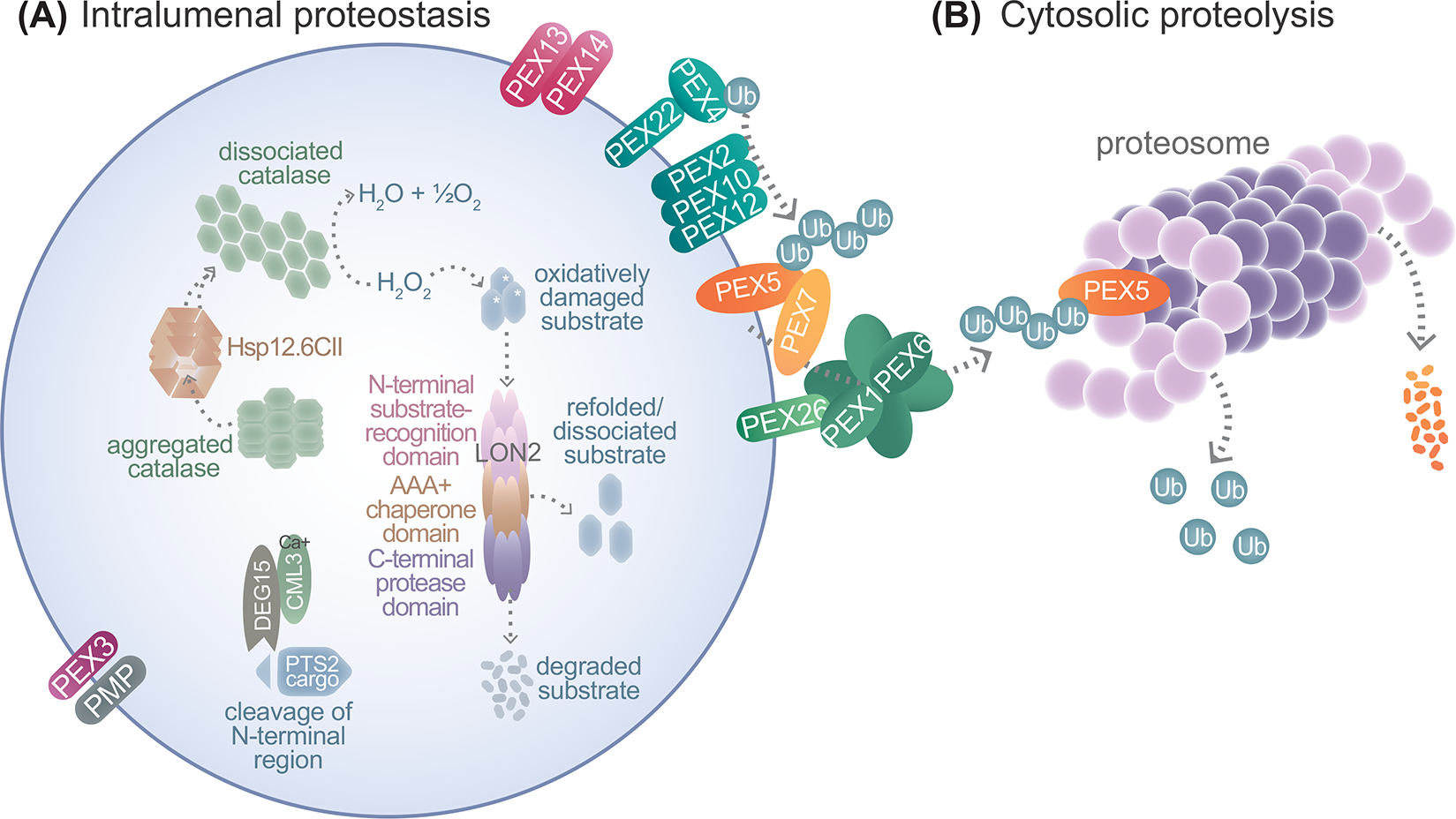
Renovating the peroxisomal proteome (**A**) Intralumenal proteostasis involves proteases and chaperones. Proteases cleave the PTS2 (peroxisome targeting signal 2) peptide and degrade damaged and obsolete proteins; chaperones assist protein folding, complex assembly, and aggregate dissociation. Peroxisomal proteins with cytosolic domains (e.g., PEX5) can be polyubiquitinated by a ubiquitin (Ub)-conjugating enzyme (PEX4) and the Ub-protein ligase complex (PEX2, PEX10, and PEX12) at the peroxisome membrane. (**B**) Polyubiquitinated proteins are degraded by the cytosolic 26S proteosome.

After proteins are incorporated into the organelle, peroxisomal proteostasis is managed by both lumenal and cytosolic machinery. Peroxisomes employ the cytosolic ubiquitin (Ub) 26S proteasome system to degrade ubiquitinated proteins to maintain peroxisomal health. The sequential action of Ub-activating enzyme (E1), Ub-conjugating enzymes (E2s), and Ub-protein ligases (E3s) covalently attach one or more ubiquitin moieties to target proteins (reviewed in [62]). Like yeast, plant peroxisomes have a dedicated E2, PEX4, that is tethered to the outside of the organelle by the PEX22 PMP [63,64]. Arabidopsis PEX4 is implicated in both PEX5 recycling and degradation [64,65] as well as degradation of a mutated PEX12 [23]. E2s work with E3s that provide substrate specificity, and many E3s are characterized by a RING domain. PEX2, PEX10, and PEX12 (Figure 1D) are RING-domain peroxins that have E3 activity *in vitro* [66]. These peroxins are implicated in both monoubiquitinating PEX5 for recycling (Figure 1D) and polyubiquitinating PEX5 for degradation by the cytosolic proteasome system (Figure 2) [23,66,67]. Furthermore, SUPPRESSOR OF PPI1 LOCUS1 (SP1), a RING-type E3 with well-documented chloroplast functions [68] may moonlight at the peroxisome to ubiquitinate the PEX13 PMP for proteasomal degradation [69], although this function is disputed [70].

Beyond PEX5 and PMPs, there is indirect evidence of ubiquitin system involvement in degrading lumenal proteins, which would require retrotranslocation of lumenal substrates out of the organelle to access the cytosolic ubiquitination machinery. For example, the lumenal proteins isocitrate lyase (ICL) and malate synthase (MLS) are stabilized in a *pex4 pex22* mutant [64,71], and a screen for mutants that stabilize a GFP-ICL fusion [72] yielded *pex2* and *pex10* alleles [73].

In addition to cytosolic quality control, the peroxisome lumen houses chaperones and proteases that have been identified through various proteomic and bioinformatic studies (Table 1). Heat shock proteins (HSPs) are molecular chaperones that regulate protein folding, assembly, and disaggregation. Chaperones also can contribute to protein degradation, and mammalian Hsp70 facilitates substrate degradation via the ubiquitin-proteasome system and autophagy pathways [74,75]. An Hsp70 with a consensus PTS2 in watermelon (*Citrullus vulgaris*) cotyledons is similar to pea (*Pisum sativum*) and cucumber (*Cucumis sativus*) plastidic Hsp70s [76]. Interestingly, cucumber peroxisomes house two Hsp70s (71 and 78 kDa) and an Hsp40 [77]. Cucumber Hsp40 is a PMP that in the ADP-bound conformation specifically binds Hsp70–1 (71 kDa), but not the heavier Hsp70–2, [77], perhaps assisting in folding and import of peroxisomal proteins.

Although apparently lacking the peroxisomal Hsp70s found in crop plants, Arabidopsis peroxisomes house several smaller heat shock proteins in the Hsp20/α-crystallin superfamily (Table 1). Hsp15.7 and Hsp17.6CII are PTS1-targeted [78–81], and ALPHA-CRYSTALLIN DOMAIN 31.2 (ACD31.2) is PTS2-targeted [80]. Both Hsp15.7 and ACD31.2 reduce non-specific protein aggregation when expressed in yeast [80]. Hsp15.7 transcripts are induced by heat stress and when catalase is inhibited [80]. Hsp17.6CII prevents CAT2 aggregation, increases CAT2 activity, and increases tolerance to abiotic stresses (Figure 2A) [79].

The nexus of protein quality control is the balance between repair and destruction—folding, assembly, or disaggregation versus breakdown for recycling. This balance is typified by the Lon family of ATP-dependent proteases, originally described in bacteria, which have both chaperone and protease functions. Arabidopsis encodes four LON isoforms; LON2 (Figure 2A) contains a PTS1 and is the only peroxisomal isoform [82,83]. LON proteases contain an N-terminal substrate-recognition domain, a central ATPase associated with various cellular activities (AAA) domain, and a C-terminal proteolytic domain (Figure 2A) [84,85]. LON N-terminal and ATPase domains together unfold misfolded proteins, contributing chaperone activity [85,86], and yeast and mammalian LONs exhibit chaperone-like activity independent of protease activity [87]. Arabidopsis *lon2* mutants display sparse, enlarged peroxisomes [88–90]. Although peroxisomal proteins are not stabilized in a *lon2* mutant [89], peroxisomal MLS and ICL (glyoxylate cycle enzymes) are synergistically stabilized in *lon2* mutants when autophagy is disabled [88,90], implicating MLS and ICL as LON2 protease substrates. Though LON2 chaperone clients are not identified in Arabidopsis, LON2 chaperone activity is necessary to prevent excessive peroxisome turnover [90].

A second protein family implicated in both proteolytic and chaperone activities is the Deg peptidase subfamily S1B proteases. *Citrullus vulgaris* GLYOXYSOMAL PROCESSING PROTEASE (GPP) and the Arabidopsis ortholog DEGRADATION OF PERIPLASMIC PROTEINS 15 (DEG15) are ATP-independent serine endopeptidases that remove the N-terminal region of PTS2 proteins *in vivo* [91]. *In vitro*, DEG15 interacts with different substrates as a monomer and dimer [92]. In the calcium-depleted monomeric state, DEG15 degrades denatured proteins. However, when bound to the peroxisomal calcium-dependent Calmodulin-like protein CML3, DEG15 specifically cleaves PTS2 proteins (Figure 2A) [92,93]. DEG15 PTS2 substrates include malate dehydrogenase, citrate synthase, acyl-CoA oxidase, and 3-ketoacyl-CoA thiolase [91,92]. PTS2 cleavage is the only DEG15 activity that is validated *in vivo*; *deg15* mutants fail to process PTS2 proteins, but reported lumenal protein levels are unaltered [91,92]. Despite failing to process PTS2 proteins, *deg15* mutants have only mild physiological defects [89], indicating that PTS2-containing enzymes are at least partially functional when uncleaved. However, the *deg15 lon2* double mutant displays synergistic defects [89], hinting that DEG15 might have roles beyond PTS2 processing. Related enzymes in bacteria also show *in vivo* chaperone activity [94–96], but whether DEG15 has proteolytic or chaperone substrates beyond PTS2 proteins remains unknown.

## Clearance of the peroxisomal proteome–pexophagy

Regulating individual proteins allows peroxisomes to tune their contents and reactions; however, plants also use more drastic measures to orchestrate large-scale organellar remodeling. The peroxisome population can be decreased via pexophagy, a selective form of autophagy, which allows for comprehensive shifts in the peroxisomal proteome (reviewed in [97]).

**Figure 3.**
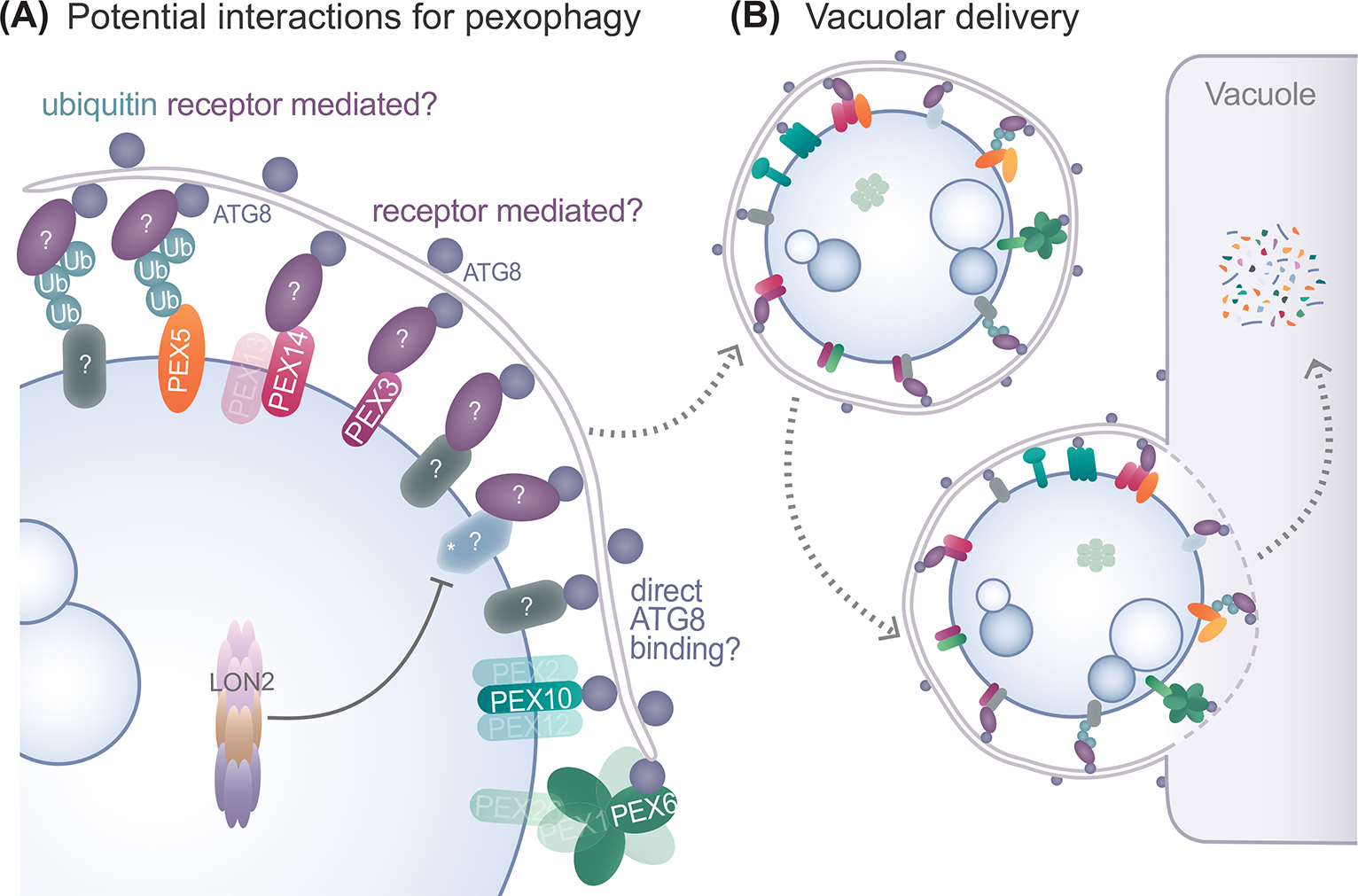
Dismantling the peroxisomal proteome (**A**) Peroxisomes are targeted for pexophagy by recruitment of autophagy machinery, including a double-bilayer isolation membrane marked by ATG8 (violet circles). Recruitment could occur through direct interaction of peroxisomal proteins with ATG8 or via an intermediary receptor (purple ovals) that recognizes peroxisomal proteins or their ubiquitinated (Ub) derivates. Illustrated targeting mechanisms are based on receptor-mediated partners observed in non-plant systems or demonstrated ATG8 interactions in plants; none of the interactions depicted have been shown as necessary or sufficient to induce pexophagy in plants. (**B**) Following recruitment of the isolation membrane, cargo is fully engulfed to form an autophagosome. Contents are delivered through fusion of the outer autophagosomal membrane with the vacuolar membrane. In the vacuole, resident proteases and lipases degrade the peroxisomal proteins and lipids to allow nutrient recycling.

During pexophagy, the entire organelle, including lumenal and membrane contents, is targeted by the autophagy machinery for disassembly in the vacuole (Figure 3), allowing the molecular components to be reused by the cell. Autophagy requires coordination of numerous autophagy-related (ATG) proteins [98]. Although the first Arabidopsis ATG genes were identified by homology in 2002 [99], pexophagy was not reported in plants until over a decade later [88,100,101]. Analysis of *atg* null mutants, which are unable to carry out either general or cargo-specific autophagy, underpins much of our knowledge of pexophagy in plants.

Pexophagy occurs even under optimal growth conditions, as indicated by elevated peroxisome abundance and protein levels in *atg* mutant seedlings [100,101]. Interestingly, proteomic analysis of Arabidopsis *atg* mutants reveals that peroxisomal proteins are more stabilized than proteins from other organelles under normal growth conditions [102], perhaps reflecting the damaging oxidative environment of the peroxisome compared to other organelles. Indeed, inactive catalase is found in aggregated peroxisomes in Arabidopsis *atg* seedlings, signifying that these peroxisomes are oxidized [100]. Moreover, stomatal opening defects are associated with decreased catalase activity and increased ROS in guard cells of *atg2* mutants [103]. Additionally, maize *atg* mutants under stress accumulate peroxisomal β-oxidation enzymes, but do not process additional lipids, suggesting that the accumulating peroxisomes may be inactive [36].

In addition to quality control, protein turnover via pexophagy supports developmental transitions in peroxisomal reactions. For example, young pumpkin seedlings are populated by numerous β-oxidative peroxisomes processing stored fats, but with the onset of photosynthesis, leaf tissue gains peroxisomes that support photorespiration [104]. Both thiolase, a β-oxidation enzyme, and MLS, a glyoxylate cycle enzyme, are stabilized in Arabidopsis *atg* mutant seedlings [88], suggesting that pexophagy plays a role in this transition.

The LON2 peroxisome-localized protease plays a regulatory role in pexophagy [88]. Intriguingly, excessive pexophagy underlies the physiological and molecular defects in *lon2* null mutants. Arabidopsis *lon2* suppressors include dozens of mutations spanning six *ATG* genes, which completely restore peroxisomal function and morphology [88,105]. A transgene expressing a protease-disabled LON2 suppresses the excessive pexophagy in *lon2*, while a version disabling the chaperone domain does not [90]. Thus, it appears that the chaperone function of LON2, rather than the peptidase function, restricts pexophagy in wild-type plants. The specific LON2 clients that promote pexophagy when LON2 is disabled remain to be identified.

Other proteins have been implicated in plant pexophagy. The actin-related proteins 2/3 (ARP2/3) complex, which nucleates actin polymerization, appears to localize to subperoxisomal surface puncta that occassionally co-localize with ATG8 in Arabidopsis [106]. Peroxisomes are metabolically functional, more abundant, and enlarged in ARP2/3 complex mutants [106], hinting at a pexophagy-related role for the complex, and by extension, actin.

Beyond LON2 and ARP2/3, our knowledge of proteins specifically involved in plant pexophagy is sparse; receptors linking peroxisomes to the autophagy machinery remain elusive. Although several pexophagy receptors are identified in yeast [107,108], these proteins lack apparent homologs in plants. In the case of the mammalian pexophagy receptor NBR1 [109], the plant homolog serves as an autophagy receptor for polyubiquitinated aggregates [110] and exocyst-positive organelles [111]. However, a role for NBR1 in mediating the excessive pexophagy observed in *lon2* mutants has been ruled out [105].

Like the pexophagy receptors, the peroxisomal proteins recognized by these receptors remain to be identified in plants. Several peroxins in the membrane are implicated in pexophagy in other systems, including PEX3 [108,112] and PEX14 [113] (Figure 3A). Moreover, the RING peroxins are positioned to ubiquitinate PMPs, which can serve as an autophagy trigger. In mammalian cells, starvation increases PEX2 expression, which promotes pexophagy via ubiquitination of peroxisomal proteins [114]. PEX5 is a pexophagy receptor ligand in ubiquitin-mediated mammalian pexophagy models (reviewed in [115,116]) (Figure 3A). Moreover, perturbing the AAA ATPase retrotranslocation complex (PEX1, PEX6, and PEX26) increases pexophagy in human cells, and this increase can be eliminated by reducing PEX5 levels [117,118], suggesting that the AAA ATPase prevents pexophagy by preventing ubiquitinated PEX5 accumulation in the membrane.

Peroxin roles in regulating pexophagy might be conserved in plants, though direct evidence remains elusive. The peroxisome-defective phenotypes of Arabidopsis *pex1* and *pex6* mutants are partially rescued by preventing autophagy [21,119]. This rescue suggests that, as in mammals [117,118], impeding the plant AAA ATPase results in autophagic targeting of peroxisomes that are at least partially functional.

One approach to discover proteins mediating pexophagy is to identify peroxisomal proteins that interact with autophagy proteins. ATG8 is a lipidated ubiquitin-like protein that marks the isolation membrane that envelops autophagy cargo to form autophagosomes [120,121]. Selective-autophagy receptors often facilitate cargo engulfment by binding ATG8 [122] via either an ATG8-interacting motif (AIM) or a ubiquitin-interacting motif (UIM)-like sequence [123]. These motifs bind at an LDS (LIR/AIM docking site) or UDS (UIM-like docking site) on ATG8 [123]. Bioinformatic analysis reveals nine Arabidopsis peroxisomal proteins with predicted AIMs, including PEX6 and PEX10, which also interact with ATG8f in biomolecular fluorescent complementation assays [124]. Moreover, PEX10 interacts with the ATG8e LDS in yeast two-hybrid and dot blot binding assays [123]. Whether PEX10 association with autophagy machinery is necessary or sufficient for pexophagy *in vivo* remains unexplored.

While precise mechanisms directing pexophagy remain elusive, identifying conditions in which pexophagy occurs can provide new avenues to study the process. Peroxisomal oxidation impacts pexophagy in multiple systems [100,125,126]. In human cells, a ROS-activated kinase promotes ROS-dependent PEX5 ubiquitination, permitting recognition by an autophagy receptor [125]. The identification of specific plant proteins mediating pexophagy will assist in determining whether the general oxidative state of the peroxisome, the state and configuration of a specific protein, or both, triggers pexophagy in plants. Other stress conditions might provide avenues to study peroxisomal proteome and population changes. For example, an ephemeral peroxisome population is induced by concerted proliferation and pexophagy during cadmium stress [127,128]. Additionally, salt induces peroxisome proliferation [129,130] and autophagy [131], but it remains unknown if selective pexophagy occurs during salt stress. It will be interesting to monitor the impact on the peroxisomal proteome and plant health as new conditions and mutants that impact the peroxisome population are identified.

## Future directions

Plant peroxisomal proteostasis requires coordination of processes from transcription to whole-organelle degradation, yet many nuclear factors that regulate these processes and protein mediators remain unidentified. Deployment of transcriptomics and proteomics using various mutants with peroxisome and autophagy dysfunction may help elucidate the interconnected processes impacting peroxisomal proteostasis. In addition, comparing data from different time points and developmental stages will illuminate the interplay of transcriptional regulation, protein function, inter-organelle communication, and responses. Such high-content datasets will provide a foundation for informative modeling studies and may also help identify substrates of the various peroxisomal proteases (Table 1), including pexophagy regulators.

The recent demonstration of peroxisome-associated translation in yeast [54,55] highlights the gaps in our knowledge of peroxisomal protein targeting and delivery in plants. Though *de novo* formation from the ER is assumed, early steps in plant peroxisome biogenesis remains largely unexplored. The role of ER trafficking in providing PMPs to budding pre-peroxisomes and pre-existing peroxisome has not been determined in plants. The gatekeeping mechanisms for membrane proteins at ER exit sites and the identity of proteins involved in addition to early-acting peroxins remains largely unknown for any organism. Interestingly, the roles for biogenesis peroxins PEX19 and PEX3 have been expanded in mammals to include ER-targeting of lipid droplet proteins, hinting at connected biogenesis [132]. Should these organelles share exit sites, findings on lipid droplet formation could provide new targets for peroxisome biogenesis and ER trafficking analysis.

The recent description of intralumenal vesicles in peroxisomes [5] prompts many questions about peroxisomal protein targeting, including possible use of these vesicles to compartmentalize PMPs, lumenal proteins, and peroxisomal metabolism. Future experiments may reveal whether PEX5, PEX11, and PEX14 occupy sub-peroxisomal domains in plants as observed in human cells [133], whether additional peroxins sort differentially, and whether this sub-organellar localization or ARP2/3 sub-domains on the peroxisomal surface [106] involves intralumenal vesicles [5].

Future experiments will undoubtedly leverage advances in CRISPR systems for targeted gene disruption and modification in plants [134]. This approach will allow studies of proteins that have previously been limited by the lack of viable mutants or the presence of duplicated genes. Moreover, CRISPR experiments allow targeting of specific functional domains. For instance, disrupting predicted AIM sequences could identify plant peroxins necessary for pexophagy. CRISPR studies will also facilitate expansion of plant peroxisome studies beyond Arabidopsis, where much of our current knowledge is based, to include other plants, including crop species.

The fluidity and adaptability of peroxisome function across development, tissues, and environments is a key attribute of the organelle. The observed versatility inspires and necessitates further exploration of how peroxisomes regulate their membrane and lumenal protein populations. Illuminating peroxisomal proteostasis remains a dynamic frontier in plant biology.

## Figures and Tables

**Table 1:** Predicted or confirmed plant peroxisomal proteases and chaperones

Identifier	Name	Alias	Class	PTS	Localization^1^

At1g28320	DEGRADATION OF PERIPLASMIC PROTEINS15	DEG15	PTS2-processing protease	PTS1	Microscopy [91]
At4g12910	SERINE CARBOXYPEPTIDASE-LIKE20	SCPL20	Serine carboxypeptidase	PTS1	Microscopy [81]
At4g36880	RD21A-LIKE PROTEASE1	RDL1	Papain-like Cys proteinase	PTS1	Microscopy [81]
At5g47040	LON PROTEASE2	LON2	Lon protease homolog	PTS1	Microscopy [82]
At2g41790	Peroxisomal M16 protease	PXM16	Zinc-metallopeptidase	PTS1	Proteomics [83]
At2g18080	EMBRYO SAC DEVELOPMENT ARREST2	EDA2	Serine carboxypeptidase	PTS1	Predicted [57]
At4g20310	SITE 2 PROTEASE	S2P	Peptidase M50 family protein	PTS2	Predicted [57]
At1g06460	ALPHA-CRYSTALLIN DOMAIN 31.2	ACD31.2	Hsp20/α-crystallin domain	PTS2	Microscopy [80]
At5g12020	17.6 kDa Class II heat shock protein	HSP17.C6II	Hsp20/α-crystallin domain	non-canonical PTS1	Microscopy [79]
At5g37670	15.7 kDa heat shock protein	HSP15.7	Hsp20/α-crystallin domain	PTS1	Microscopy [80]
*Cucumis sativus* Q04960	DnaJ peptide-binding protein	HSP40	J-domain-containing protein	?	Proteomics [135]
*Citrullus lanatus* U92815	Heat shock protein 70	HSP70	70-kDa heat shock protein	PTS2	Proteomics [76]
*Cucumis sativus* AJ249330	Heat shock protein 70	HSP70-1	70-kDa heat shock protein	?	Proteomics [77]
*Cucumis sativus* AJ249331	Heat shock protein 70	HSP70-2	70-kDa heat shock protein	?	Proteomics [77]

1 Localizations are based on the presence of a PTS (predicted), from peroxisome proteomic studies (proteomics), or confirmed using fusion to fluorescent reporters (microscopy).

## Summary

- Peroxisomes alter their proteome in profound ways to support plant vitality based on tissue, condition, and developmental stage.
- Recent discoveries suggest that peroxisomes share more attributes with other organelles than previously appreciated, such as protein sorting, membrane complexity, and localized translation.
- Despite important contributions to our understanding of peroxisomal proteostasis, high-throughput analyses are a largely untapped reservoir for future studies elucidating additional facets of peroxisomal proteostasis.

## Abbreviations

AAA: ATPase associated with various cellular activities
ABI5: ABA (abscisic acid) insensitive 5
AIM: ATG8-interacting motif
ATG: autophagy-related
CAT: catalase
CML: calmodulin-like
CRISPR: clustered regularly interspaced short palindromic repeats
ER: endoplasmic reticulum
GBF1: G-BOX binding factor 1
HSP: heat shock protein
HYH: HY5-homolog
ICL: isocitrate lyase
ILV: intralumenal vesicle
LDS: LIR/AIM-docking site
LIR: LC3-interacting region (LC3 is a mammalian ATG8 ortholog)
MAPK: mitogen-activated protein kinase
MLS: malate synthase
PEX: peroxin
PHY: phytochrome
PMP: peroxisomal membrane protein
PTS: peroxisome-targeting signal
RING: really interesting new gene
ROS: reactive oxygen species
UDS: UIM-like docking site
UIM: ubiquitin-interacting motif
